# First genome sequencing and comparative analyses of *Corynebacterium pseudotuberculosis* strains from Mexico

**DOI:** 10.1186/s40793-018-0325-z

**Published:** 2018-10-10

**Authors:** Doglas Parise, Mariana T D Parise, Marcus V C Viana, Adrian V Muñoz-Bucio, Yazmin A Cortés-Pérez, Beatriz Arellano-Reynoso, Efrén Díaz-Aparicio, Fernanda A Dorella, Felipe L Pereira, Alex F Carvalho, Henrique C P Figueiredo, Preetam Ghosh, Debmalya Barh, Anne C P Gomide, Vasco A C Azevedo

**Affiliations:** 1 Laboratory of Cellular and Molecular Genetics, Institute of Biologic Sciences, Federal University of Minas Gerais, Belo Horizonte, MG Brazil; 2 Department of Microbiology and Immunology, Faculty of Veterinary Medicine and Zootechnics, National Autonomous University of Mexico, Mexico City, Mexico; 3 Aquacen - National Reference Laboratory for Aquatic Animal Diseases, Federal University of Minas Gerais, Belo Horizonte, MG Brazil; 4 Department of Computer Science, Virginia Commonwealth University, Richmond, VA-23284 USA; 5 Centre for Genomics and Applied Gene Technology, Institute of Integrative Omics and Applied Biotechnology (IIOAB), Nonakuri, Purba Medinipur, West Bengal 721172 India; 6 Division of Bioinformatics and Computational Genomics, NITTE University Center for Science Education and Research (NUCSER), NITTE (Deemed to be University), Deralakatte, Mangaluru, Karnataka India

**Keywords:** Phylogenetics, Genomic sequencing, Drug target, CRISPR-Cas, Restriction-modification systems

## Abstract

*Corynebacterium pseudotuberculosis* is a pathogenic bacterium which has been rapidly spreading all over the world, causing economic losses in the agricultural sector and sporadically infecting humans. Six *C. pseudotuberculosis* strains were isolated from goats, sheep, and horses with distinct abscess locations. For the first time, Mexican genomes of this bacterium were sequenced and studied in silico. All strains were sequenced using Ion Personal Genome Machine sequencer, assembled using Newbler and SPAdes software. The automatic genome annotation was done using the software RAST and in-house scripts for transference, followed by manual curation using Artemis software and BLAST against NCBI and UniProt databases. The six genomes are publicly available in NCBI database. The analysis of nucleotide sequence similarity and the generated phylogenetic tree led to the observation that the Mexican strains are more similar between strains from the same host, but the genetic structure is probably more influenced by transportation of animals between farms than host preference. Also, a putative drug target was predicted and in silico analysis of 46 strains showed two gene clusters capable of differentiating the biovars *equi* and *ovis*: Restriction Modification system and CRISPR-Cas cluster.

**Electronic supplementary material:**

The online version of this article (10.1186/s40793-018-0325-z) contains supplementary material, which is available to authorized users.

## Introduction

*Corynebacterium pseudotuberculosis* is a Gram-positive bacterium that infects several different species of mammals. Strains of the biovar *ovis* infect sheep and goats, and strains of the biovar *equi* infect larger mammals such as horses, camels, and buffaloes. The manifestation of the infection depends on the host [1–4]. This bacterium causes significant economic loss to animal production all over the world due to reduced production of wool, milk and meat, carcass condemnation, as well as the death of infected animals [4–6]. *C. pseudotuberculosis* can also affect humans, causing distinct kinds of lymphadenitis. Contamination occurs through contact with infected animals and consumption of infected food [4, 5, 7].

This organism affects several countries such as Australia, Brazil, Canada, Egypt, Israel, New Zealand, South Africa, United Kingdom and United States [4, 8–17]. Cases in other countries such as Portugal [18], Mexico [19] and Equatorial Guinea [20] have been reported in the recent years. In the United States, *C. pseudotuberculosis* infections are reemerging and considered endemic [19], and the state with the highest number of cases of this bacterium was Texas, which borders Mexico [21]. The spread of *C. pseudotuberculosis* to other countries brings out the importance of improving the understanding of this bacterium. In the present study, six Mexican *C. pseudotuberculosis* strains were investigated, two from the biovar *equi* and four from the biovar *ovis*. This is the first time that strains of this bacterium, isolated in Mexico, have been completely sequenced. Among those strains, these are the first isolates of the biovar *equi* coming from this country [19]. The characterization of these strains is important for achieving a better understanding of this species, considering they can present relevant features not yet identified in other strains.

## Organism information

*C. pseudotuberculosis* is a pathogenic bacterium that belongs to the CMNR (*Corynebacterium*, *Mycobacterium*, *Nocardia**,* and *Rhodococcus*) group. This group is characterized by high GC content (46–74%) and by the structure of the cell wall which is mainly composed of peptidoglycan, arabinogalactan and mycolic acids [4, 22]. *C. pseudotuberculosis* is placed in the phylum *Actinobacteria*, class *Actinobacteria*, order *Actinomycetales*, suborder *Corynebacterineae* and genus *Corynebacterium* [23–30]. The species is considered a facultative intracellular pathogen [4, 31] which is Gram-positive, pleomorphic, non-motile, non-sporulating, mesophilic and can survive both in the host and in the soil [25, 31–35]. Its strains are classified into two biovars, *ovis* and *equi*, according to its host preference and nitrate reduction capacity, which is identified through the presence or absence of the *narG* gene in a PCR Multiplex test [36]. The biovar *equi* can reduce nitrate and affects mostly large ruminants. The biovar *ovis* is not able to reduce nitrate and affects mostly small ruminants [4]. More information about classification, general features of this species and some details about the target strains are shown in Table 1 (Additional file 1).

**Table 1:** Classification and general features of *Corynebacterium* strains MEX1, MEX9, MEX25, MEX29, MEX30, and MEX31 according to the MIGS recommendations [41]

MIGS ID	Property	Term	Evidence code^a^
	Classification	Domain *Bacteria*	TAS [23]
		Phylum *Actinobacteria*	TAS [24]
		Class *Actinobacteria*	TAS [25]
		Order *Actinomycetales* *Suborder* *Corynebacterineae*	TAS [25–28]
		Family *Corynebacteriaceae*	TAS [25, 28]
		Genus *Corynebacterium*	TAS [29, 30]
		Species *Corynebacterium pseudotuberculosis*	TAS [26, 29]
		strain: *MEX1 (Accession NZ_CP017711.1)* *MEX9 (Accession NZ_CP014543.1),* *MEX25* *(Accession NZ_CP013697.1),* *MEX29* *(Accession NZ_CP016826.1),* *MEX30* *(Accession NZ_CP017291.1),* *MEX31* *(Accession NZ_CP017292.1)*	
	Gram stain	*Positive*	TAS [31]
	Cell shape	*Pleomorphic*	TAS [31]
	Motility	*Non-motile*	TAS [31, 35]
	Sporulation	*non-sporulating*	TAS [31]
	Temperature range	*Mesophilic*	TAS [32, 35]
	Optimum temperature	*37 °C*	TAS [32, 73]
	pH range; Optimum	*7.0–7.2*	TAS [4, 35]
	Carbon source	*Glucose, fructose, maltose, mannose, and sucrose*	TAS [11, 15]
MIGS-6	Habitat	*Host and soil*	TAS [25, 33, 34]
MIGS-6.3	Salinity	*Up to 2 M NaCl*	TAS [32]
MIGS-22	Oxygen requirement	*Aerobic and facultative anaerobic*	TAS [4, 35, 73]
MIGS-15	Biotic relationship	*Facultative intracellular pathogen*	TAS [4, 31]
MIGS-14	Pathogenicity	*Sheep, goats, horses, cattle, camel, buffalo, rarely humans*	TAS [4, 37, 74]
MIGS-4	Geographic location	*MEX1 – Ixtenco, Tlaxcala, Mexico* *MEX9 – Salamanca, Guanajuato, Mexico* *MEX25* *– Celaya, Guanajuato, Mexico* *MEX29* *- Río Frio, Estado de Mexico, Mexico* *MEX30* *and* *MEX31* *– Valparaiso, Zacatecas, Mexico*	TAS [19]
MIGS-5	Sample collection	*MEX1–2014, MEX9 and* *MEX25* *–2012,* *MEX29* *,* *MEX30* *and* *MEX31* *–2013*	TAS [19]
MIGS-4.1	Latitude	*MEX1 – 19* ^*o*^ *15’11” MEX9–20* ^*o*^ *34’26”* *MEX25* *–20* ^*o*^ *55’1”* *MEX29* *–19* ^*o*^ *21’8”* *MEX30* *and* *MEX31* *–22* ^*o*^ *46’16”*	IDA
MIGS-4.2	Longitude	*MEX1 – 97* ^*o*^ *53’45” MEX9 - 101* ^*o*^ *11’45”* *MEX25* *- 101* ^*o*^ *9’42”* *MEX29* *- 98* ^*o*^ *40’17”* *MEX30* *and* *MEX31* *–103* ^*o*^ *34’11”*	IDA
MIGS-4.4	Altitude	*MEX1–8236 ft MEX9–5623 ft* *MEX25* *–6502 ft* *MEX29* *–9770 ft* *MEX30* *and* *MEX31* *–6221 ft*	IDA

^a^Evidence codes - IDA: Inferred from Direct Assay; TAS: Traceable Author Statement (i.e., a direct report exists in the literature); NAS: Non-traceable Author Statement (i.e., not directly observed for the living, isolated sample, but based on a generally accepted property for the species, or anecdotal evidence). These evidence codes are from the Gene Ontology project [75]

Six *C. pseudotuberculosis* strains were isolated in Mexico from different hosts and biovars. The strain MEX1 was isolated from a retropharyngeal abscess in a goat. The strain MEX9 was isolated from a prescapular abscess in a goat. The strain MEX25 was isolated from a parotidean abscess in a sheep. The strain MEX29 was isolated from a retropharyngeal abscess in a sheep. These four strains presented negative result for the presence of the *narG* gene in the PCR multiplex test and were classified as belonging to the biovar *ovis*. All *ovis* strains were obtained from outbreaks occurred relatively close to Mexico City. MEX30 and MEX31 were isolated from abscesses in the pectoral muscles of two horses [19]. These two strains were positive for the presence of the *narG* gene in PCR Multiplex. Consequently, they were classified as belonging to the biovar *equi*. Although both *equi* strains were obtained in the same city, they could be considered as isolated cases.

**Fig. 1:**
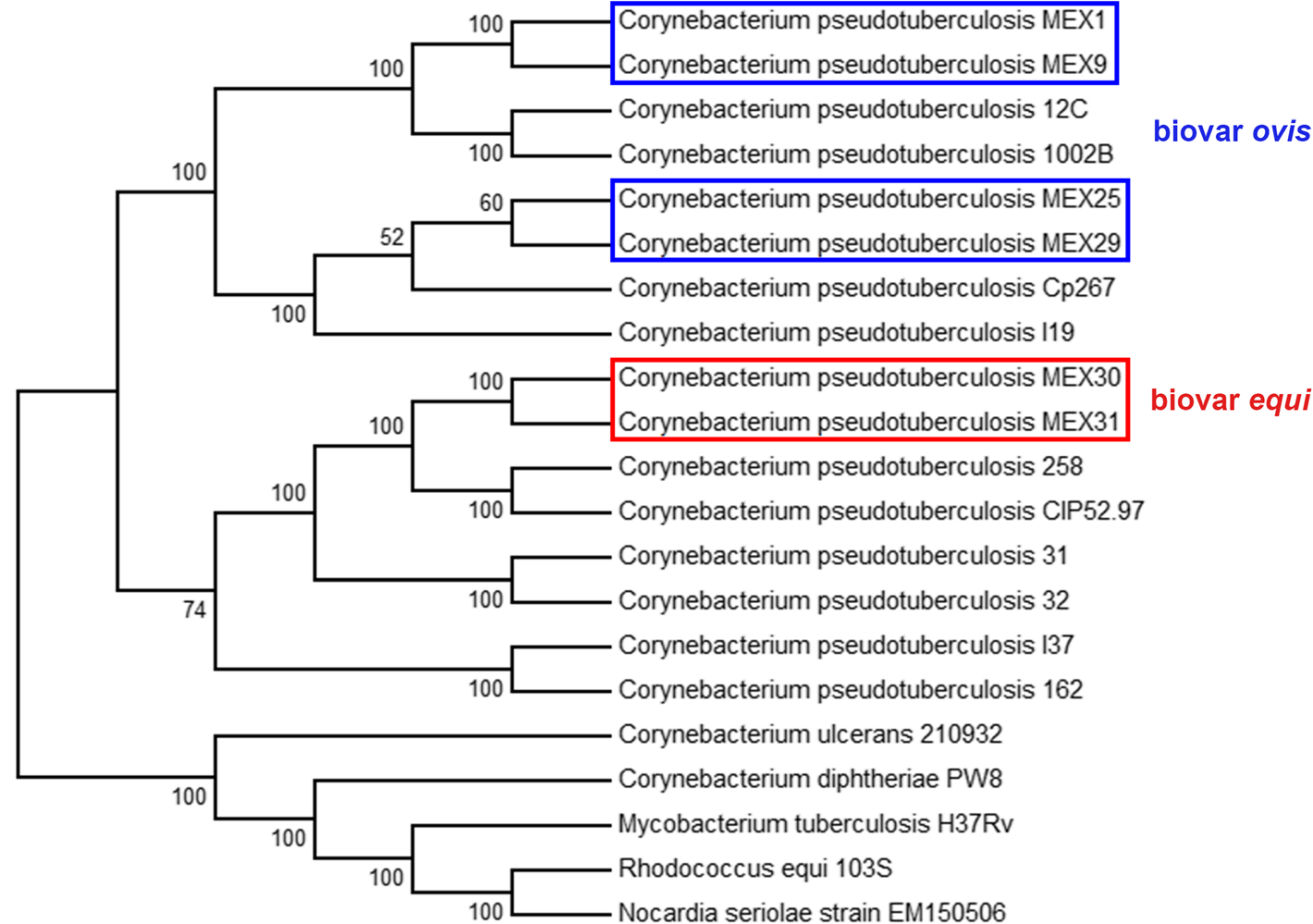
Phylogenetic tree of new *Corynebacterium pseudotuberculosis* strains of this work inside the rectangles, with other strains of the group CMNR. The blue rectangles highlight the biovar *ovis* strains and the red rectangle highlights the biovar *equi* strains of this work. The numbers near the nodes indicate bootstrap values

To verify the phylogenetic relationship of these strains to other strains of *C. pseudotuberculosis*, we generated a phylogenetic tree (Fig. 1) based on the core proteome and progressive refinement, using a bootstrap value of 100. The tree was generated using the PEPR software (https://github.com/enordber/pepr.git) with the Maximum-Likelihood method. The Mexican strains were clustered according to the respective biovars and host preferences, as shown in previous works) [1, 37].

MEX30 and MEX31 were isolated in Valparaiso, in the first reported case of infection of horses in Mexico [19]. They clustered together probably because they came from the same source, that could be transported infected animals. Affected horses were identified in all regions of the US and the state of Texas, which borders Mexico, has the highest number of cases) [9, 21].

Ovis strains were isolated in Tlaxcala (MEX1) and Rio Frio de Juárez (MEX29), with a 50 Km distance from each other, and Guanajuato (MEX9 and MEX25), within a 400–450 Km distance from the two other isolation localities. However, the strains cluster by host rather than locality of isolation. MEX1 and MEX9 were isolated from goat and MEX25 and MEX29 were isolated from sheep. However, MEX25 and MEX29 (goat) clustered with isolates from lhama (USA) and cow (Israel), while MEX1 and MEX9 (sheep) clustered with isolates from goat and sheep (Brazil), all with a 100% bootstrap. Strains of Ovis biovar are more clonal but does not show the same degree of clustering by the host as Equi [1, 37]. Considering a maximum distance of 450 Km between localities of isolation, this genetic structure could better be explained by farming history than host preference. The goat and sheep farms could have different sources of Ovis strains. Transportation of infected animals and further contact and transmission of the disease probably occurred between farms of the same host species [38–40].

## Genome sequencing information

### Genome project history

The present project is a collaboration between the National Autonomous University of Mexico (UNAM), Mexico City, Mexico, and the Federal University of Minas Gerais (UFMG), Belo Horizonte, Minas Gerais, Brazil. The six *C. pseudotuberculosis* strains were isolated by UNAM researchers. Sequencing was performed at the National Reference Laboratory for Aquatic Animal Diseases (AQUACEN), and the two processes of assembly and annotation were performed at the Laboratory of Cellular and Molecular Genetics (LGCM), both laboratories located at UFMG. All genomes are complete and available at the National Center for Biotechnology Information (NCBI). This information is shown in Table 2 and conforms with MIGS recommendations [41]. As mentioned above, the present study presents the first sequencing of *C. pseudotuberculosis*, and the first isolation of the biovar *equi*, from Mexico. This data can provide new insights into the diagnosis and treatment of diseases caused by this organism.

**Table 2:** Project information

MIGS ID	Property	Term
MIGS 31	Finishing quality	Finished
MIGS-28	Libraries used	Fragments
MIGS 29	Sequencing platforms	Ion Torrent PGM
MIGS 31.2	Fold coverage	115× (MEX1); 129× (MEX9); 99× (MEX25); 135× (MEX29); 81× (MEX30); 123× (MEX31).
MIGS 30	Assemblers	Newbler, SPAdes.
MIGS 32	Gene calling method	RAST
	Locus Tag	CpMEX1_ (MEX1); CpMEX9_ (MEX9); AN397_ (MEX25); CpMEX29_ (MEX29); CpMEX30_ (MEX30); CpMEX31_ (MEX31);
	Genbank ID	CP017711 (MEX1); CP014543(MEX9); CP013697 (MEX25); CP016826 (MEX29); CP017291 (MEX30); CP017292 (MEX31);
	GenBank Date of Release	2017/01/30 (MEX1); 2016/05/27 (MEX9); 2015/12/23 (MEX25); 2016/11/03 (MEX29); 2016/12/27 (MEX30); 2016/12/27 (MEX1);
	GOLD ID	- (MEX1); Go0366057 (MEX9); Go0139540 (MEX25); Go0364114 (MEX29); Go0364489 (MEX30); Go0364678 (MEX31);
	BIOPROJECT	PRJNA348354 (MEX1); PRJNA312392 (MEX9); PRJNA294672 (MEX25); PRJNA335634 (MEX29); PRJNA343017 (MEX30); PRJNA341961 (MEX31);
MIGS 13	Source Material Identifier	BHI broth
	Project relevance	Animal Pathogen, Medical

### Growth conditions and genomic DNA preparation

The samples used in the present study are in the sample collection of LGCM. All six strains were grown in a brain-heart-infusion media (BHI-HiMedia Laboratories Pvt. Ltd., India) with 1.5% of bacteriological agar and supplemented with 0.5% of Tween 80, at 37 °C for 72 h under rotation. Genomic DNA was extracted following the protocol of Pacheco et al. [36].

### Genome sequencing and assembly

The first step in sequencing each genome was the library construction, following manufacturer’s recommendations (IonXpress™ Plus gDNA Fragment Library Preparation). This was performed in three steps: (i) DNA fragmentation using the Ion Shear™ Plus Reagents Kit, (ii) addition of adapters using Ion Xpress™ Barcode Adapters and (iii) library amplification using the Ion PGM™ Template OT2 200 kit (all kits from Thermo Fisher Scientific, USA). The resulting library was put on the semiconductor chip Ion 318 Chip Kit v2 (Thermo Fisher Scientific) and then into the sequencer Ion Personal Genome Machine™ (Thermo Fisher Scientific). The number of reads and the mean read length of MEX1, MEX9, MEX25, MEX29, MEX30 and MEX31 strains are respectively: 1,100,551 and 244; 1,496,261 and 201; 1,117,243 and 206; 1,371,907 and 230; 1,127,325 and 186; and, 1,262,316 and 230.

The assembly process was managed using SIMBA software [42]. The quality assessment of the reads was performed using FastQC software [43]. The assemblies were performed using SPAdes version 3.6 [44] on MEX1 and MEX31; and, Newbler version 2.9 (Roche, USA) on MEX9, MEX25, MEX29, and MEX30. This produced the following contigs: 6 on MEX1, 7 on MEX9, 7 on MEX25, 9 on MEX29, 33 on MEX30 and 13 on MEX31. The N50 s were: 543,202 on MEX1, 372,309 on MEX9, 543,326 on MEX25, 367,275 on MEX29, 103,276 on MEX30 and 535,978 on MEX31. The QUAST software [45] was used to evaluate the quality of the assemblies for all strains. The scaffolds were constructed using CONTIGuator software version 2.0 [46] with *C. pseudotuberculosis* strain 29,156 (CP010795.1) as a reference to MEX9, MEX25 and MEX29, *C. pseudotuberculosis* strain MEX9 as a reference to MEX1, *C. pseudotuberculosis* strain 316 (CP003077.1) as a reference to MEX30 and *C. pseudotuberculosis* strain E19 (CP012136.1) as a reference to MEX31. Gap closure was performed using CLC Genomics Workbench 7 (Qiagen, USA). This process resulted in six complete genome sequences.

### Genome annotation

Genome annotation was performed in two steps: automatic annotation and manual curation. The RAST [47] and tRNAscan-SE [48] software were used in the automated annotation. An in-house script was also employed to transfer the annotation from a reference genome. The Artemis software version 16.0.0 [49], the UniProt [50] and the National Center for Biotechnology Information (NCBI) databases [51] were used in the manual curation. Putative frameshifts were analyzed using CLC Genomics Workbench 7 (Qiagen, USA) and fixed whenever possible.

## Genome properties

**Fig. 2:**
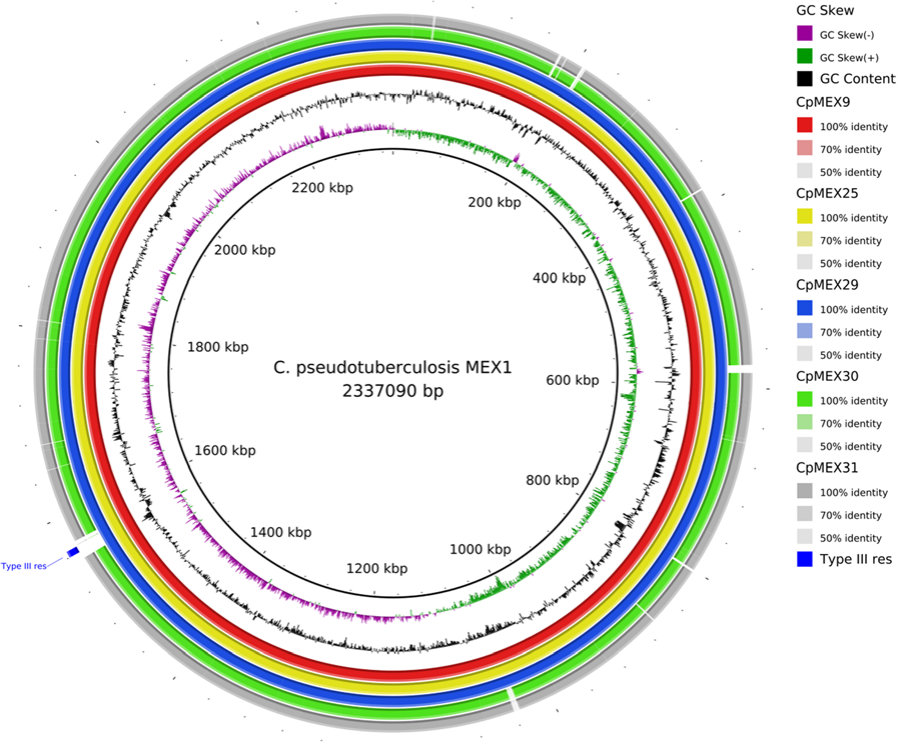
Circular map of *C. pseudotuberculosis* strain MEX1 (biovar *ovis*) in comparison with the other strains of this study. The cluster of methylation type III, which is only present in biovar *ovis* strains, is highlighted in blue

**Fig. 3:**
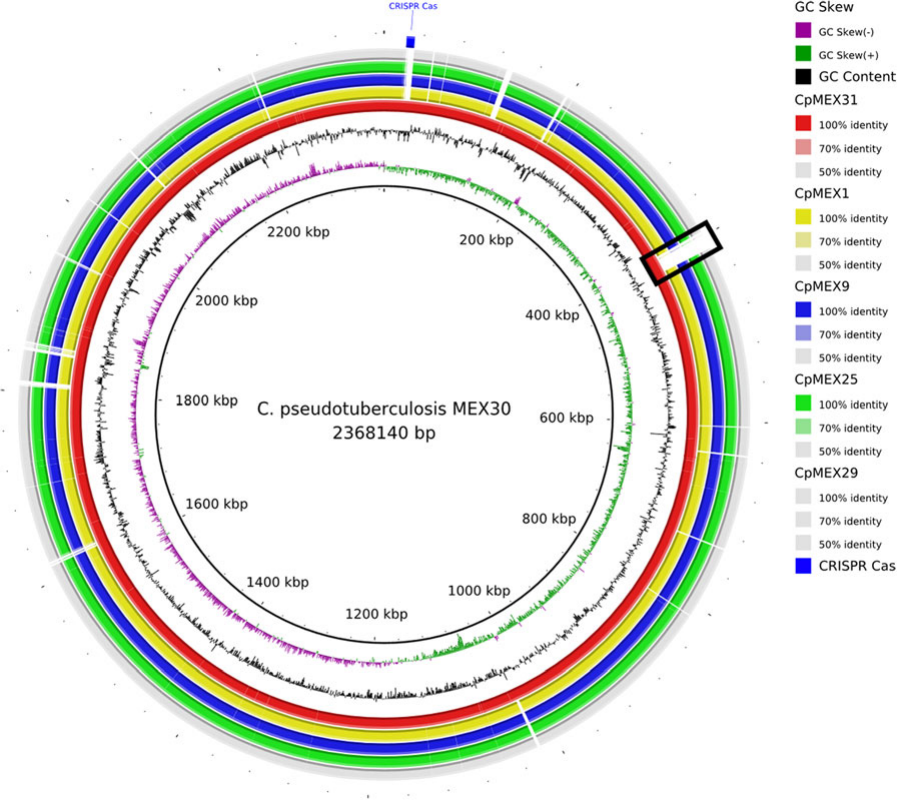
Circular map of *C. pseudotuberculosis* strain MEX30 (biovar *equi*) in comparison with the other strains of this study. The cluster of CRISPR-Cas, which is only present in biovar *equi* strains, is highlighted in blue. The nitrate reductase gene cluster is highlighted by a black rectangle

Genome sizes of the respective strains are: 2,337,090 bp (base pairs) on MEX1, 2,337,578 bp on MEX9, 2,337,529 bp on MEX25, 2,337,866 bp on MEX29, 2,368,140 bp on MEX30 and 2,367,880 bp on MEX31. The respective percentages of the predicted coding regions are: 86.16% on MEX1, 86.33% on MEX9, 85.94% on MEX25, 86.66% on MEX29, 83.06% on MEX30 and 86.64% on MEX31. These genome sizes and the G + C content (~ 52%) are consistent with other *C. pseudotuberculosis* studies [2, 6, 52]. There are 64 predicted RNA genes in strains of the biovar *ovis* (MEX1, MEX9, MEX25 and MEX29) and 66 from the biovar *equi* (MEX30 and MEX31). The numbers (and percentages) of predicted protein coding genes and pseudogenes of MEX1, MEX9, MEX25, MEX29, MEX30 and MEX31 strains are, respectively: 2021 (94.22%) and 60 (2.80%); 2025 (94.36%) and 57 (2.66%); 2016 (94.07%) and 63 (2.94%); 2032 (94.73%) and 49 (2.28%); 2008 (91.77%) and 114 (5.21%); and 2058 (94.32%) and 61 (2.80%). Table 3 shows detailed information about properties and statistics of these genomes. The number of genes associated with general COG functional categories [53, 54] was generated with the in-house script Blast Cog (https://github.com/aquacen/blast_cog) and are summarized in Table 4. The circular maps of *C. pseudotuberculosis* MEX1 and MEX30 strains in comparison with the other strains of the present study are shown in Figs. 2 and 3, respectively.

**Table 3:** Genome statistics

Attribute	MEX1		MEX9		MEX25		MEX29		MEX30		MEX31	
	Value	%	Value	%	Value	%	Value	%	Value	%	Value	%
Genome size (bp)	2,337,090	100.0	2,337,578	100.0	2,337,529	100.0	2,337,866	100.0	2,368,140	100.0	2,367,880	100.0
DNA coding (bp)	2,012,758	86.12	2,017,915	86.33	2008,915	85.94	2025,972	86.66	1,966,942	83.06	2,051,473	86.64
DNA G + C (bp)	1,219,520	52.18	1,219,842	52.18	1,219,763	52.18	1,219,957	52.18	1,234,064	52.11	1,233,547	52.10
DNA scaffolds	1	100.0	1	100.0	1	100.0	1	100.0	1	100.0	1	100.0
Total genes	2145	100.0	2146	100.0	2143	100.0	2145	100.0	2188	100.0	2182	100.0
Protein coding genes	2021	94.22	2025	94.36	2016	94.07	2032	94.73	2008	91.77	2058	94.32
RNA genes	64	2.98	64	2.98	64	2.99	64	2.98	66	3.02	63	2.89
Pseudo genes	60	2.80	57	2.66	63	2.94	49	2.28	114	5.21	61	2.80
Genes in internal clusters	NA	NA	NA	NA	NA	NA	NA	NA	NA	NA	NA	NA
Genes with function prediction	1579	73.61	1576	73.44	1583	73.87	1578	73.57	1605	73.36	1610	73.79
Genes assigned to COGs	2007	93.57	2013	93.80	2009	93.75	2020	94.17	1998	91.32	2046	93.77
Genes with Pfam domains	1679	78.28	1675	78.05	1670	77.93	1690	78.79	1664	76.05	1731	79.33
Genes with signal peptides	157	7.32	151	7.04	159	7.42	155	7.23	144	6.58	153	7.01
Genes with transmembrane helices	595	27.74	591	27.54	585	27.30	601	28.02	585	26.74	596	27.31
CRISPR repeats	0	00 0	1	0.05	1	0.05	1	0.05	4	0.18	4	0.18

**Table 4:** Number of genes associated with general COG functional categories

Code	MEX1		MEX9		MEX25		MEX29		MEX30		MEX31		Description
	Value	%age	Value	%age	Value	%age	Value	%age	Value	%age	Value	%age	
J	182	9.01	182	8.99	181	8.98	186	9.15	183	9.11	189	9.18	Translation, ribosomal structure and biogenesis
A	2	0.10	2	0.10	2	0.10	2	0.10	2	0.10	2	0.10	RNA processing and modification
K	138	6.83	139	6.87	137	6.80	138	6.79	136	6.77	134	6.51	Transcription
L	105	5.20	105	5.19	96	4.76	102	5.02	102	5.08	101	4.91	Replication, recombination and repair
B	0	0	0	0	0	0	0	0	1	0.05	0	0	Chromatin structure and dynamics
D	44	2.18	43	2.12	45	2.23	45	2.22	43	2.14	47	2.28	Cell cycle control, Cell division, chromosome partitioning
V	68	3.37	67	3.31	69	3.42	71	3.49	75	3.74	70	3.40	Defense mechanisms
T	99	4.90	101	4.99	99	4.91	103	5.07	98	4.88	98	4.76	Signal transduction mechanisms
M	124	6.14	122	6.03	119	5.90	120	5.91	119	5.93	117	5.69	Cell wall/membrane biogenesis
N	21	1.04	22	1.09	20	0.99	20	0.98	13	0.65	17	0.83	Cell motility
U	32	1.58	31	1.53	30	1.49	31	1.53	29	1.44	30	1.46	Intracellular trafficking and secretion
O	128	6.33	122	6.03	121	6.00	126	6.20	122	6.08	122	5.93	Posttranslational modification, protein turnover, chaperones
C	125	6.19	124	6.12	116	5.75	124	6.10	123	6.13	121	5.88	Energy production and conversion
G	158	7.82	154	7.61	151	7.49	156	7.68	151	7.52	161	7.82	Carbohydrate transport and metabolism
E	212	10.49	213	10.52	204	10.12	213	10.48	219	10.91	223	10.84	Amino acid transport and metabolism
F	82	4.06	82	4.05	79	3.92	82	4.04	78	3.88	81	3.94	Nucleotide transport and metabolism
H	143	7.08	141	6.96	135	6.70	141	6.94	151	7.52	152	7.39	Coenzyme transport and metabolism
I	92	4.55	91	4.49	90	4.46	93	4.58	86	4.28	87	4.23	Lipid transport and metabolism
P	162	8.02	157	7.75	162	8.04	162	7.97	166	8.27	168	8.16	Inorganic ion transport and metabolism
Q	49	2.43	46	2.27	46	2.28	48	2.36	52	2.59	50	2.43	Secondary metabolites biosynthesis, transport, and catabolism
R	170	8.41	164	8.10	157	7.79	167	8.22	167	8.32	169	8.21	General function prediction only
S	138	6.83	142	7.01	127	6.30	141	6.94	138	6.87	140	6.80	Function unknown
–	14	0.69	12	0.59	7	0.35	12	0.59	10	0.50	12	0.58	Not in COGs

The total is based on the total number of protein coding genes in the genome

## Insights from the genome sequence

**Fig. 4:**
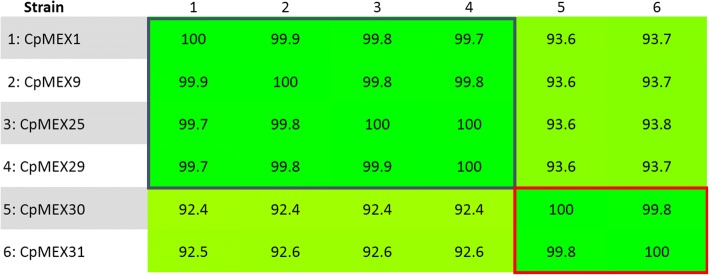
Alignment generated using Gegenees software showing the percentage similarity among the strains, based on the accessory genome. The blue rectangle highlights the grouping of the biovar *ovis*. The red rectangle highlights the grouping of the biovar *equi*

The nucleotide sequences, analyzed using the Gegenees software version 2.1 [55], show high similarity (> 92%) between the strains. Higher similarity (> = 99.7%) within strains belonging to the same biovar was found (Fig. 4). This is consistent with a previous study [1], using 15 strains of *C. pseudotuberculosis*, that shows similarity greater than 99% within the biovar *ovis* strains and at least 95% of sequencing similarity within the biovar *equi* strains. Moreover, the sequencing similarity among strains isolated from the same host is higher than the similarity among strains isolated from different hosts (Figs. 1 and 4).

Traditionally, the two biovars are differentiated using a nitrate reduction test, in which *equi* is positive, and *ovis* is negative [56]. Figure 3 highlights the cluster of genes related to nitrate reduction in Mexican *equi* strains with the black rectangle. The Protein Family Sorter tool [57] was used to search for genes or clusters of genes that may be used to differentiate the biovars. Within the six genomes of the present study, we found the cluster of genes that is related to proteins of type III restriction-modification (RM) systems [58, 59] exclusively in the biovar *ovis* (highlighted in blue in Fig. 2). A cluster of genes related to the proteins of Clustered Regularly Interspaced Short Palindromic Repeats (CRISPR-Cas) systems, probably belonging to type I-E [60], was found exclusively in the biovar *equi* (highlighted in blue in Fig. 3). Both systems work as protection systems, defending the bacteria against exogenous DNA. We analyzed 40 other sequenced strains of *C. pseudotuberculosis* to confirm these results in other strains. The same pattern was observed.

RM systems have two main components, a DNA methyltransferase, and a restriction endonuclease. The first one methylates the DNA in possible cleavage sites; the second one is responsible for the cleavage of DNA from external sources [61]. A good review of RM systems can be found in [62]. CRISPR-Cas systems are adaptive immune systems in bacteria and archaea. They use a complex of proteins known as Cas that are responsible for acquiring new, short sequences of external sources (exogenous genetic elements). These short sequences are incorporated into the bacterial chromosome and are called CRISPRs. The CRISPRs are transcribed into small RNAs that guide the Cas proteins to recognize and cleave foreign DNA, protecting the bacterial genome [63]. Reviews of CRISPR-Cas systems can be found in [63–65].

Possible new drug targets were predicted using the Specialty Genes Search from the Pathosystems Resource Integration Center (PATRIC) bioinformatics resource center [66]. The result shows a new putative target, the gene *nrdF2*, for five of the six strains used in the present study. In the *C. pseudotuberculosis* MEX30 strain, this gene is annotated as a pseudogene, which can explain why it was not considered a putative target. The product of this gene is the small subunit of ribonucleotide reductase (RNR) which is involved in dNTP (deoxynucleotide triphosphate) synthesis that reduces ribonucleotides to nucleotides. The RNRs can be classified into three classes (I, II and III). Class I is oxygen dependent and has two subclasses (Ia and Ib). Class Ia is coded by *nrdA* and *nrdB* genes; class Ib is coded by *nrdE* and *nrdF*. Therefore, the RNR found in the biovar *ovis* strains belongs to class Ib [67]. Previous studies [68–70] show the importance of this gene for growth under normal conditions (in vitro) in *Mycobacterium tuberculosis*, *Corynebacterium ammoniagenes* and *Corynebacterium glutamicum*. Additionally, other studies have pointed to this gene as a potential target of *M. tuberculosis* vaccine [70–72].

## Conclusions

In the present study, we investigated six strains of *C. pseudotuberculosis* from different hosts and their sequenced genomes, the first whole-genome investigation of this organism from Mexico. The phylogenomic analysis suggested that the genetic structure of Ovis is more influenced by animal transportation than host preference. An in silico analysis of protein families showed two important clusters that may differentiate the biovars *equi* and *ovis*. Also, the present work identified a new putative drug target against *C. pseudotuberculosis*, the gene *nrdF2*, which has been previously described as a potential vaccine target [70–72]. Further in silico and in vitro analyses are required to validate these findings. Those results could provide a better understanding of this organism and its mechanisms of virulence and pathogenesis, as well as develop new diagnoses, vaccines, and treatments.

## Additional file

Additional file 1: Contains tables Annotation Summary, GenBank Accession Summary, Strain ID Summary, Plant Name Summary, Scientific Name Summary and Reference Search Summary. (DOCX 17 kb)

## Abbreviations

AQUACEN: National Reference Laboratory for Aquatic Animal Diseases
BHI: Brain heart infusion
CRISPR: Clustered regularly interspaced short palindromic repeats
LGMC: Laboratory of Cellular and Molecular Genetics
PATRIC: Pathosystems Resource Integration Center
PEPR: Phylogenomic Estimation with Progressive Refinement
RM systems: Restriction-modification
UFMG: Federal University of Minas Gerais
UNAM: National Autonomous University of Mexico
