# Supplementary material for: First genome sequencing and comparative analyses of Corynebacterium pseudotuberculosis strains from Mexico
Source: Stand Genomic Sci. 2018 Oct 10;13:21. doi: 10.1186/s40793-018-0325-z (PMC6180578; doi:10.1186/s40793-018-0325-z)
Supplement: Supplementary file 1 — Contains tables Annotation Summary, GenBank Accession Summary, Strain ID Summary, Plant Name Summary, Scientific Name Summary and Reference Search Summary. (DOCX 17 kb) [file 40793_2018_325_MOESM1_ESM.docx]

**Annotation Summary**

| Name | Count | References | Comments |
| --- | --- | --- | --- |
| Actinobacteria | 6 | PHYLUM Actinobacteria:  M. Goodfellow: Phylum XXVI. Actinobacteria phyl. nov. Bergey's Manual of Systematic Bacteriology 2012, 5, Part A: 33.  CLASS Actinobacteria:  E. Stackebrandt, F.A. Rainey, N.L. Ward-Rainey: Proposal for a new hierarchic classification system, Actinobacteria classis nov. International Journal of Systematic Bacteriology 1997, 47: 479-491. | 'Actinobacteria' resolved to multiple names (homonyms):  <https://doi.org/10.1601/nm.5711>  phylum Actinobacteria  <https://doi.org/10.1601/nm.5712>  class Actinobacteria |
| Actinomycetales | 2 | ORDER Actinomycetales:  R.E. Buchanan: Studies in the nomenclature and classification of bacteria. II. The primary subdivisions of the Schizomycetes. Journal of Bacteriology 1917, 2: 155-164.  V.B.D. Skerman, V. McGowan, P.H.A. Sneath: Approved Lists of Bacterial Names. International Journal of Systematic Bacteriology 1980, 30: 225-420. |  |
| Brevibacterium stationis | 1 | SPECIES Brevibacterium stationis:  R.S. Breed: The Brevibacteriaceae fam. nov. of order Eubacteriales. Riassunti della Communicazione, VI Congresso Internazionale di Microbiologia, Roma 1953, 1: 13-14.  V.B.D. Skerman, V. McGowan, P.H.A. Sneath: Approved Lists of Bacterial Names. International Journal of Systematic Bacteriology 1980, 30: 225-420. |  |
| Corynebacteriaceae | 2 | FAMILY Corynebacteriaceae:  K.B. Lehmann, R. Neumann: Lehmann's Medizin, Handatlanten. X Atlas und Grundriss der Bakteriologie und Lehrbuch der speziellen bakteriologischen Diagnostik. Lehmann's Medizin, Handatlanten. X Atlas und Grundriss der Bakteriologie und Lehrbuch der speziellen bakteriologischen Diagnostik. 1907, 2: 270.  V.B.D. Skerman, V. McGowan, P.H.A. Sneath: Approved Lists of Bacterial Names. International Journal of Systematic Bacteriology 1980, 30: 225-420. |  |
| Corynebacterineae | 2 | SUBORDER Corynebacterineae:  E. Stackebrandt, F.A. Rainey, N.L. Ward-Rainey: Proposal for a new hierarchic classification system, Actinobacteria classis nov. International Journal of Systematic Bacteriology 1997, 47: 479-491. |  |
| Corynebacterium | 8 | GENUS Corynebacterium:  K.B. Lehmann, R. Neumann: Atlas und Grundriss der Bakteriologie und Lehrbuch der speziellen bakteriologischen Diagnostik. Atlas und Grundriss der Bakteriologie und Lehrbuch der speziellen bakteriologischen Diagnostik 1896, 1-448.  V.B.D. Skerman, V. McGowan, P.H.A. Sneath: Approved Lists of Bacterial Names. International Journal of Systematic Bacteriology 1980, 30: 225-420. |  |
| Corynebacterium ammoniagenes | 2 | SPECIES Corynebacterium ammoniagenes:  M.D. Collins: Transfer of Brevibacterium ammoniagenes, Cooke and Keith to the genus Corynebacterium as Corynebacterium ammoniagenes comb. nov. International Journal of Systematic Bacteriology 1987, 37: 442-443. |  |
| Corynebacterium diphtheriae | 1 | SPECIES Corynebacterium diphtheriae:  K.B. Lehmann, R. Neumann: Atlas und Grundriss der Bakteriologie und Lehrbuch der speziellen bakteriologischen Diagnostik. Atlas und Grundriss der Bakteriologie und Lehrbuch der speziellen bakteriologischen Diagnostik 1896, 1-448.  V.B.D. Skerman, V. McGowan, P.H.A. Sneath: Approved Lists of Bacterial Names. International Journal of Systematic Bacteriology 1980, 30: 225-420. |  |
| Corynebacterium glutamicum | 2 | SPECIES Corynebacterium glutamicum:  S. Abe, K. Takayama, S. Kinoshita: Taxonomic studies on glutamic acid-producing bacteria. The Journal of General and Applied Microbiology 1967, 13: 279-301.  V.B.D. Skerman, V. McGowan, P.H.A. Sneath: Approved Lists of Bacterial Names. International Journal of Systematic Bacteriology 1980, 30: 225-420. |  |
| Corynebacterium pseudotuberculosis | 54 | SPECIES Corynebacterium pseudotuberculosis:  F. Eberson: A bacteriologic study of the diphtheroid organisms with special reference to Hodgkin's disease. The Journal of Infectious Diseases 1918, 23: 1-42.  V.B.D. Skerman, V. McGowan, P.H.A. Sneath: Approved Lists of Bacterial Names. International Journal of Systematic Bacteriology 1980, 30: 225-420. |  |
| Corynebacterium stationis | 1 | SPECIES Corynebacterium stationis:  K.A. Bernard, D. Wiebe, T. Burdz, A. Reimer, B. Ng, C. Singh, S. Schindle, A.L. Pacheco: Assignment of Brevibacterium stationis (ZoBell and Upham 1944) Breed 1953 to the genus Corynebacterium, as Corynebacterium stationis comb. nov., and emended description of the genus Corynebacterium to include isolates that can alkalinize citrate. International Journal of Systematic and Evolutionary Microbiology 2010, 60: 874-879. |  |
| Mycobacterium | 1 | GENUS Mycobacterium:  K.B. Lehmann, R. Neumann: Atlas und Grundriss der Bakteriologie und Lehrbuch der speziellen bakteriologischen Diagnostik. Atlas und Grundriss der Bakteriologie und Lehrbuch der speziellen bakteriologischen Diagnostik 1896, 1-448.  V.B.D. Skerman, V. McGowan, P.H.A. Sneath: Approved Lists of Bacterial Names. International Journal of Systematic Bacteriology 1980, 30: 225-420. |  |
| Mycobacterium smegmatis | 1 | SPECIES Mycobacterium smegmatis:  K.B. Lehmann, R. Neumann: a. Lehmann's Medizin, Handatlanten X. Atlas und Grundriss der Bakteriologie und Lehrbuch der speziellen bakteriologischen Diagnostik 1899, 1-497.  V.B.D. Skerman, V. McGowan, P.H.A. Sneath: Approved Lists of Bacterial Names. International Journal of Systematic Bacteriology 1980, 30: 225-420. |  |
| Mycobacterium tuberculosis | 5 | SPECIES Mycobacterium tuberculosis:  K.B. Lehmann, R. Neumann: Atlas und Grundriss der Bakteriologie und Lehrbuch der speziellen bakteriologischen Diagnostik. Atlas und Grundriss der Bakteriologie und Lehrbuch der speziellen bakteriologischen Diagnostik 1896, 1-448.  V.B.D. Skerman, V. McGowan, P.H.A. Sneath: Approved Lists of Bacterial Names. International Journal of Systematic Bacteriology 1980, 30: 225-420. |  |
| Neisseria gonorrhoeae | 1 | SPECIES Neisseria gonorrhoeae:  V.B.D. Skerman, V. McGowan, P.H.A. Sneath: Approved Lists of Bacterial Names. International Journal of Systematic Bacteriology 1980, 30: 225-420.  V. Trevisan: Carratteri di alcuni nuovi generi di Batteriacee. Atti della Accademia Fisica-Medica-Statistica in Milano, Series 4 1885, 3: 92-107. |  |
| Neisseria meningitidis | 1 | SPECIES Neisseria meningitidis:  E.G.D. Murray: The meningococcus. Special report series (Medical Research Council (Great Britain)) 1929, 124: 7-142.  V.B.D. Skerman, V. McGowan, P.H.A. Sneath: Approved Lists of Bacterial Names. International Journal of Systematic Bacteriology 1980, 30: 225-420. |  |
| Nocardia | 1 | GENUS Nocardia:  V. Trevisan: I. Generi e le Specie delle Batteriacee. I. Generi e le Specie delle Batteriacee 1889, 563-569.  V.B.D. Skerman, V. McGowan, P.H.A. Sneath: Approved Lists of Bacterial Names. International Journal of Systematic Bacteriology 1980, 30: 225-420. |  |
| Rhodococcus | 1 | GENUS Rhodococcus:  V.B.D. Skerman, V. McGowan, P.H.A. Sneath: Approved Lists of Bacterial Names. International Journal of Systematic Bacteriology 1980, 30: 225-420.  W. Zopf: Über Ausscheidung von Fettfarbstoffen (Lipochromen) seitens gewisser Spaltpilze. Berichte der Deutschen Botanischen Gesellschaft 1891, 9: 22-28. |  |

**GenBank Accession Summary**

| GenBank Accession | Summary |
| --- | --- |
| PRJNA343017 | BioProject Accession: https://www.ncbi.nlm.nih.gov/bioproject/PRJNA343017 |
| CP012136 | CP012136.1 is a bacterial sequences record containing circular, double-stranded DNA (2,367,956 bases) from Corynebacterium pseudotuberculosis strain E19. It contains 4,355 features, including 2182 genes with 1214 distinct annotations, 2112 DNA coding regions, 12 rRNA features (5S ribosomal RNA, 16S ribosomal RNA and 23S ribosomal RNA) and 48 tRNA features coding for 19 distinct amino acids. The record was created on July 24, 2015 and last updated December 9, 2015. |
| CP014543 | CP014543.1 is a bacterial sequences record containing circular, double-stranded DNA (2,337,578 bases) from Corynebacterium pseudotuberculosis strain MEX9. It contains 4,304 features, including 2175 genes with 1217 distinct annotations, 2067 DNA coding regions, 12 rRNA features (5S ribosomal RNA, 16S ribosomal RNA and 23S ribosomal RNA) and 49 tRNA features coding for 20 distinct amino acids. The record was created on May 27, 2016. |
| CP017291 | CP017291.1 is a bacterial sequences record containing circular, double-stranded DNA (2,368,140 bases) from Corynebacterium pseudotuberculosis strain MEX 30. It contains 4,325 features, including 2208 genes with 1234 distinct annotations, 2053 DNA coding regions, 12 rRNA features (5S ribosomal RNA, 16S ribosomal RNA and 23S ribosomal RNA) and 51 tRNA features coding for 20 distinct amino acids. The record was created on December 27, 2016. |
| PRJNA312392 | BioProject Accession: https://www.ncbi.nlm.nih.gov/bioproject/PRJNA312392 |
| CP017292 | CP017292.1 is a bacterial sequences record containing circular, double-stranded DNA (2,367,880 bases) from Corynebacterium pseudotuberculosis strain MEX 31. It contains 4,344 features, including 2202 genes with 1242 distinct annotations, 2081 DNA coding regions, 12 rRNA features (5S ribosomal RNA, 16S ribosomal RNA and 23S ribosomal RNA) and 48 tRNA features coding for 19 distinct amino acids. The record was created on December 27, 2016. |
| PRJNA335634 | BioProject Accession: https://www.ncbi.nlm.nih.gov/bioproject/PRJNA335634 |
| CP016826 | CP016826.1 is a bacterial sequences record containing circular, double-stranded DNA (2,337,866 bases) from Corynebacterium pseudotuberculosis strain MEX 29. It contains 4,313 features, including 2173 genes with 1225 distinct annotations, 2078 DNA coding regions, 12 rRNA features (5S RNA, Small Subunit Ribosomal RNA; ssuRNA; SSU ribosomal RNA, 5S ribosomal RNA and Large Subunit Ribosomal RNA; lsuRNA; LSU ribosomal RNA) and 49 tRNA features coding for 20 distinct amino acids. The record was created on November 3, 2016. |
| CP003077 | CP003077.1 is a bacterial sequences record containing circular, double-stranded DNA (2,310,415 bases) from Corynebacterium pseudotuberculosis strain 316. It contains 4,402 features, including 2234 genes with 1173 distinct annotations, 2106 DNA coding regions, 12 rRNA features (5S ribosomal RNA, 16S ribosomal RNA and 23S ribosomal RNA) and 49 tRNA features coding for 20 distinct amino acids. The record was created on February 22, 2012 and last updated January 31, 2014. |
| PRJNA348354 | BioProject Accession: https://www.ncbi.nlm.nih.gov/bioproject/PRJNA348354 |
| PRJNA294672 | BioProject Accession: https://www.ncbi.nlm.nih.gov/bioproject/PRJNA294672 |
| PRJNA341961 | BioProject Accession: https://www.ncbi.nlm.nih.gov/bioproject/PRJNA341961 |
| CP013697 | CP013697.1 is a bacterial sequences record containing circular, double-stranded DNA (2,337,529 bases) from Corynebacterium pseudotuberculosis strain MEX 25. It contains 4,154 features, including 2099 genes with 44 distinct annotations, 1992 DNA coding regions, 12 rRNA features (5S ribosomal RNA, 16S ribosomal RNA and 23S ribosomal RNA) and 49 tRNA features coding for 20 distinct amino acids. The record was created on December 23, 2015. |
| CP010795 | CP010795.1 is a bacterial sequences record containing circular, double-stranded DNA (2,338,645 bases) from Corynebacterium pseudotuberculosis strain 29156. It contains 4,344 features, including 2192 genes with 1175 distinct annotations, 2089 DNA coding regions, 12 rRNA features (5S ribosomal RNA, 16S ribosomal RNA and 23S ribosomal RNA) and 50 tRNA features coding for 21 distinct amino acids. The record was created on June 3, 2015. |

**Strain ID Summary**

| Strain ID | Summary |
| --- | --- |
| ATCC 13032 | Collection Code: ATCC  Collection Name: American Type Culture Collection  Institution: ATCC (American Type Culture Collection) - United States  Strain ID: ATCC 13032 |
